# Assessing the feasibility of mapping the tibialis anterior muscle with navigated transcranial magnetic stimulation in neuro-oncologic patients

**DOI:** 10.1038/s41598-022-23444-x

**Published:** 2022-11-04

**Authors:** Thomas Eibl, Michael Schrey, Jens Weigel, Adrian Liebert, Rüdiger Lange, Michael Städt, Florian Eff, Markus Holtmannspötter, Hans-Herbert Steiner

**Affiliations:** 1 Department of Neurosurgery, Paracelsus Medical University, Breslauer Str. 201, 90471 Nuremberg, Bavaria Germany; 2 Department of Neurology, Paracelsus Medical University, Breslauer Str. 201, 90471 Nuremberg, Bavaria Germany; 3 Section Neuroradiology, Department of Radiology, Paracelsus Medical University, Breslauer Str. 201, 90471 Nuremberg, Bavaria Germany

**Keywords:** Neurology, Surgical oncology, CNS cancer

## Abstract

Mapping the lower extremity with navigated transcranial magnetic stimulation (nTMS) still remains challenging for the investigator. Clinical factors influencing leg mapping with nTMS have not been fully investigated yet. The aim of the study was to identify factors which influence the possibility of eliciting motor evoked potentials (MEPs) from the tibialis anterior muscle (TA). Patient records, imaging, nTMS examinations and tractography were retrospectively evaluated. 48 nTMS examinations were performed in 46 brain tumor patients. Reproducible MEPs were recorded in 20 patients (41.67%). Younger age (*p* = 0.044) and absence of perifocal edema (*p* = 0.035, Cramer’s V = 0.34, OR = 0.22, 95% CI = 0.06–0.81) facilitated mapping the TA muscle. Leg motor deficit (*p* = 0.49, Cramer’s V = 0.12, OR = 0.53, 95%CI = 0.12–2.36), tumor entity (*p* = 0.36, Cramer’s V = 0.22), tumor location (*p* = 0.52, Cramer’s V = 0.26) and stimulation intensity (*p* = 0.158) were no significant factors. The distance between the tumor and the pyramidal tract was higher (*p* = 0.005) in patients with successful mapping of the TA. The possibility to stimulate the leg motor area was associated with no postoperative aggravation of motor deficits in general (*p* = 0.005, Cramer’s V = 0.45, OR = 0.63, 95%CI = 0.46–0.85) but could not serve as a specific predictor of postoperative lower extremity function. In conclusion, successful mapping of the TA muscle for neurosurgical planning is influenced by young patient age, absence of edema and greater distance to the CST, whereas tumor entity and stimulation intensity were non-significant.

## Introduction

Since its introduction to neurosurgery in the first decade of the twenty-first century^1^, navigated transcranial magnetic stimulation (nTMS) has become an emerging diagnostic tool in the preoperative course of brain tumor patients. Navigated transcranial magnetic stimulation can guide decision making towards or against surgery and leads to improved outcomes concerning extent of resection compared to patients who did not receive nTMS prior to brain tumor surgery^2–4^.

Apart from the influence of nTMS on the extent of resection and neurological postoperative status, other factors influencing the success of presurgical nTMS mappings have been investigated^5,6^. Results of nTMS mapping should allow a risk stratification for tumor resection and nTMS data should also be suitable to perform a tailored approach in craniotomy to maintain the patient’s motor function. Preoperative information concerning the cortical representation of the leg area is of utmost interest before resection of motor-eloquent lesions close to the midline or interhemispheric fissure.

Investigating the feasibility of eliciting motor evoked potentials (MEPs) from different muscle groups, the anatomical constitutions need to be considered firstly. The area of the anatomic hand motor-hotspot can easily be recognized due to its omega-like shape^7,8^, whereas the anatomic leg motor-hotspot is located in the parasagittal cortical zone^7,9–12^. Previous studies found strong evidence for a very high correlation of nTMS data concerning the location of motor eloquent areas compared to intraoperative mapping so that we can assume a very high positive predictive value concerning the spatial location of nTMS positive sites^13–15^.

nTMS mapping of the leg area is still challenging and not always possible, even in experienced nTMS users^15–17^. The anatomic location of the leg motor hotspot parasagittal near the interhemispheric fissure, anatomic distortion by a tumor or extensive peritumoral edema might increase the difficulty of mapping the leg area even more.

Since mapping the leg motor area with nTMS is obviously more difficult than mapping the upper limb muscles, we wanted to answer the question if there are certain clinical conditions which further increase this difficulty.

Several clinical circumstances and nTMS examination parameters which influence the results of nTMS examinations of the upper extremity muscles have been investigated and reported^5,6,8,18,19^.

Distinct factors underlying the feasibility of mapping the leg area have not been completely investigated yet.

We hypothesized that there are distinct clinical and patient-individual factors as well as nTMS-specific conditions such as stimulation parameters and user-dependency which aggravate the difficulty in eliciting motor evoked potentials from the lower limb in brain tumor patients apart from anatomical considerations as mentioned above.

This is why we evaluated factors which contribute to successful mapping the tibialis anterior (TA) muscle, which is a widely mapped muscle in nTMS studies^5,7,17,20–22^, with nTMS in the present study.

## Methods

### Ethical standard

The study design was approved by the Institutional Review Board of Paracelsus Medical University Nuremberg (IRB-2020-022) and the study was conducted in accordance with the Declaration of Helsinki and its later amendments. Informed consent was waived due to retrospective data collection and anonymized statistical analysis.

### Study design

We present a retrospective single institution study. 46 adult patients with brain tumors who received nTMS mappings of the affected hemisphere for surgical planning were included between April 2016 and September 2020. Two patients were examined twice, for primary and recurrent tumor resection. Data from patient records, imaging and nTMS examinations were evaluated.

### nTMS mapping protocol

The nTMS examinations were performed following standardized protocol and in accordance with published recommendations^20^.

All mapping examinations were performed with the Nexstim NBS 5 System (Nexstim Oy, Helsinki, Finland) with the system’s standard figure-of-eight-coil and a biphasic stimulation pulse. The examinations were performed by two board-certified neurosurgeons trained by the system’s manufacturer or a medical student under direct supervision of one the above-mentioned neurosurgeons.

A 3D T1-post-contrast MRI scan (slice thickness 1 mm) was used as a reference imaging in each patient. Surface electrodes (Neuroline 720, Ambu, Denmark) were attached to the monitored muscles for continuous EMG-monitoring. The ground electrode was placed to the patient’s elbow. The standard protocol required monitoring of two short hand muscles and the tibialis anterior (TA) muscle. Further muscles were mapped according to the examiner’s preferences. For our present analysis, only responses from the TA muscle were taken into account.

After co-registration of the patient’s head with the 3D-MRI-scan, motor mapping was conducted and is described as follows.

**Figure 1:**
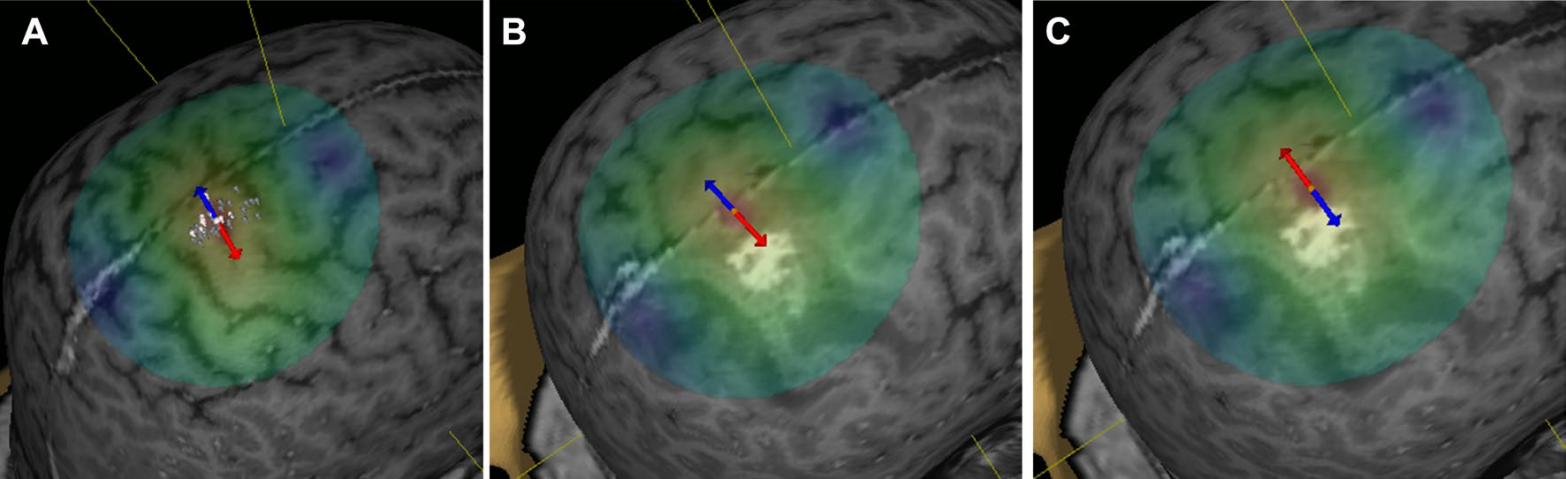
(**A**) Positive and negative stimulations within the leg area in a patient with Glioblastoma. Positive responses are indicated in white and negative stimulations are marked in grey. The electric field orientation is shown as the red and blue arrow. (**B**, **C**) Orientation of the stimulation coil during mapping of the leg motor area of a different patient. Electric field orientation with the main orientation towards the interhemispheric fissure (blue arrow lateral) and towards the hemisphere of interest (red arrow lateral).

The first step was to perform a rough mapping of the primary motor areas of each patients to discover the hand motor hotspot. Second, the patient-individual resting motor threshold (rMT) was determined at the hand motor hotspot for one upper extremity muscle. The resting motor threshold was estimated with the system’s inbuilt rMT-determination-algorithm. For mapping of the leg motor area, the coil was orientated perpendicular to the interhemispheric fissure (Fig. 1A–C). The electric field orientation towards or away from the interhemispheric fissure was orientated away from the interhemispheric fissure (i.e. towards the hemisphere of interest) as a default setting and the orientation of the electric field was changed by 180 degrees towards the interhemispheric fissure in some examinations as a choice of the nTMS examiner according to the examination conditions. Stimulations were applied to the precentral gyrus close to the interhemispheric fissure and the adjacent gyri according to the tumor location. The stimulation coil was moved a few millimeters in anterior–posterior direction or laterally after each stimulation in order to determine the spot to elicit MEPs or subsequently to create a motor map. The mean intensity at which leg mapping was started, was 131.03%rMT ranging from 105.7 to 200%rMT. The choice of the start intensity was according to the examination conditions, resting motor threshold and the examiner’s preferences. The stimulation intensity was increased according to the examination conditions, feasibility to elicit MEPs and if tolerated by the patient. The intensity was altered every 15–20 stimulations but alterations in intensity did not follow a strict protocol. The amount of increase in stimulation intensity was upon the decision of the nTMS examiner. The mean increase in stimulation intensity was 23.25%rMT per increase. The patients were instructed to have their muscles relaxed. In cases of many baseline artifacts, the examination was paused and the patient was instructed to move the targeted extremity and to relax again. In cases of no MEPs from the TA muscle, pre-activation was not performed. Each nTMS examination was evaluated post-hoc by the first author. Mapping of the leg area was considered possible if at least one MEP greater than 50 µV could be registered in the anatomic leg area.

In the following, mapping of the TA muscle was defined as successful or reproducible if it was possible to elicit more than one MEP from the TA muscle (i.e. to reproducibly elicit MEPs). Examinations with one single MEP from the TA or no MEPs were classified as not successful.

Positive and negative responses of the TA muscle, applied stimulation intensities, average amplitudes and latencies were recorded as well as if the orientation of the electric field was towards or away from the targeted hemisphere (Fig. 1B,C). In order to account for a wide range of stimulation intensities, applied stimulations were divided into subsections according to the relative stimulation intensity in % of the patient-individual rMT of the upper extremity leading to separate stimulation counts at intensities below 120%rMT, 120–130%rMT, 130–140%rMT, continued up to intensities greater than 200%rMT. Applied stimulations and MEP counts were recorded for each intensity range.

### Imaging data and tractography analysis

All patients received a MRI scan with a 1.5 T or 3.0 T scanner (Philips Healthcare, The Netherlands) with post-contrast 3D-sequences and—upon the operating surgeon’s demand—diffusion tensor imaging (DTI) with 32 diffusion gradient directions and a voxel size of 2 × 2 × 2 mm^3^.

Tumor volume (in cm^3^) was measured using the Medtronic StealthStation S8® (Medtronic Inc. Louisville, CO, USA).

**Figure 2:**
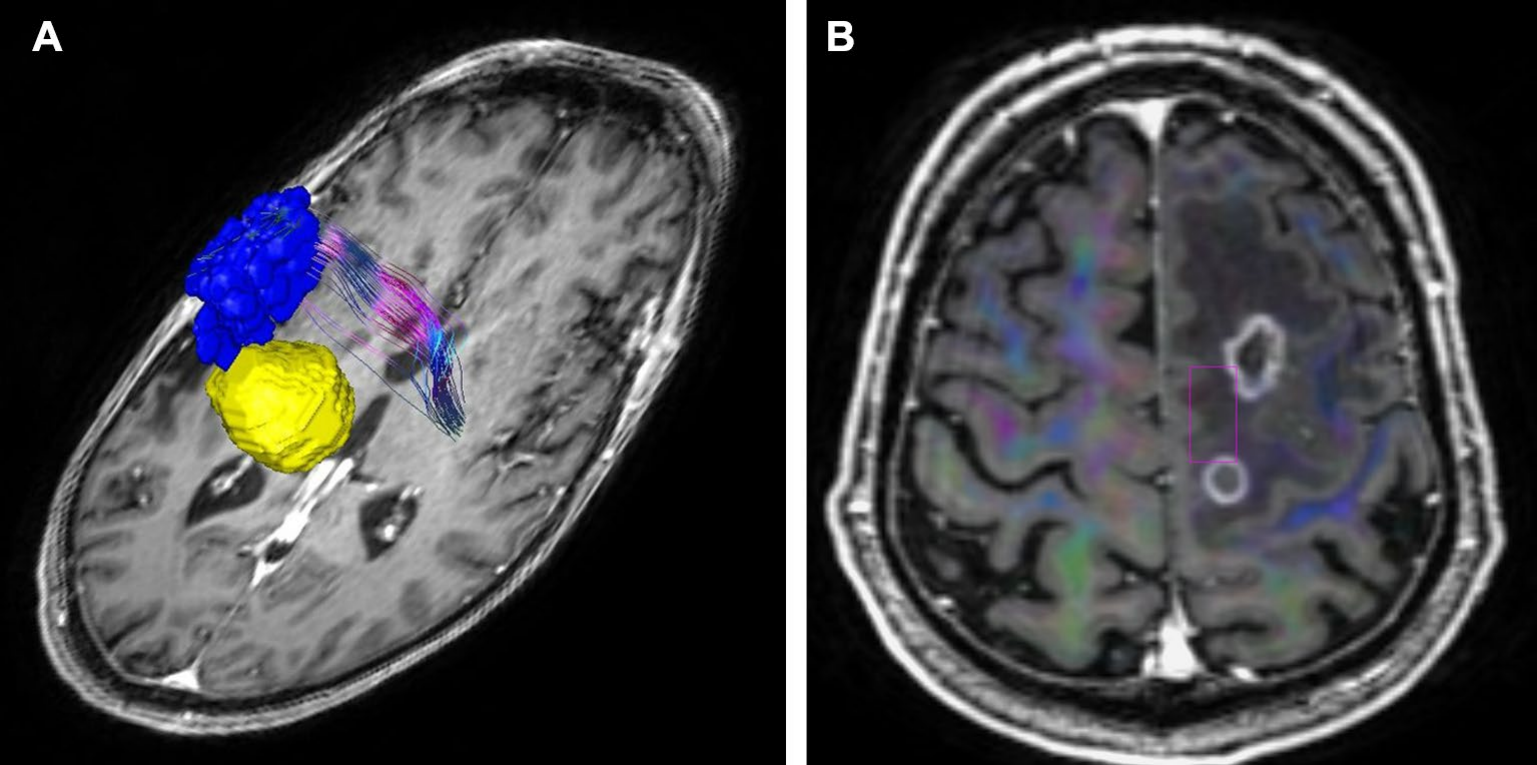
(**A**) nTMS based ROI (blue) creation and nTMS based reconstruction of the CST. The tumor is displayed in yellow. (**B**) Anatomical ROI seeding (pink).

**Figure 3:**
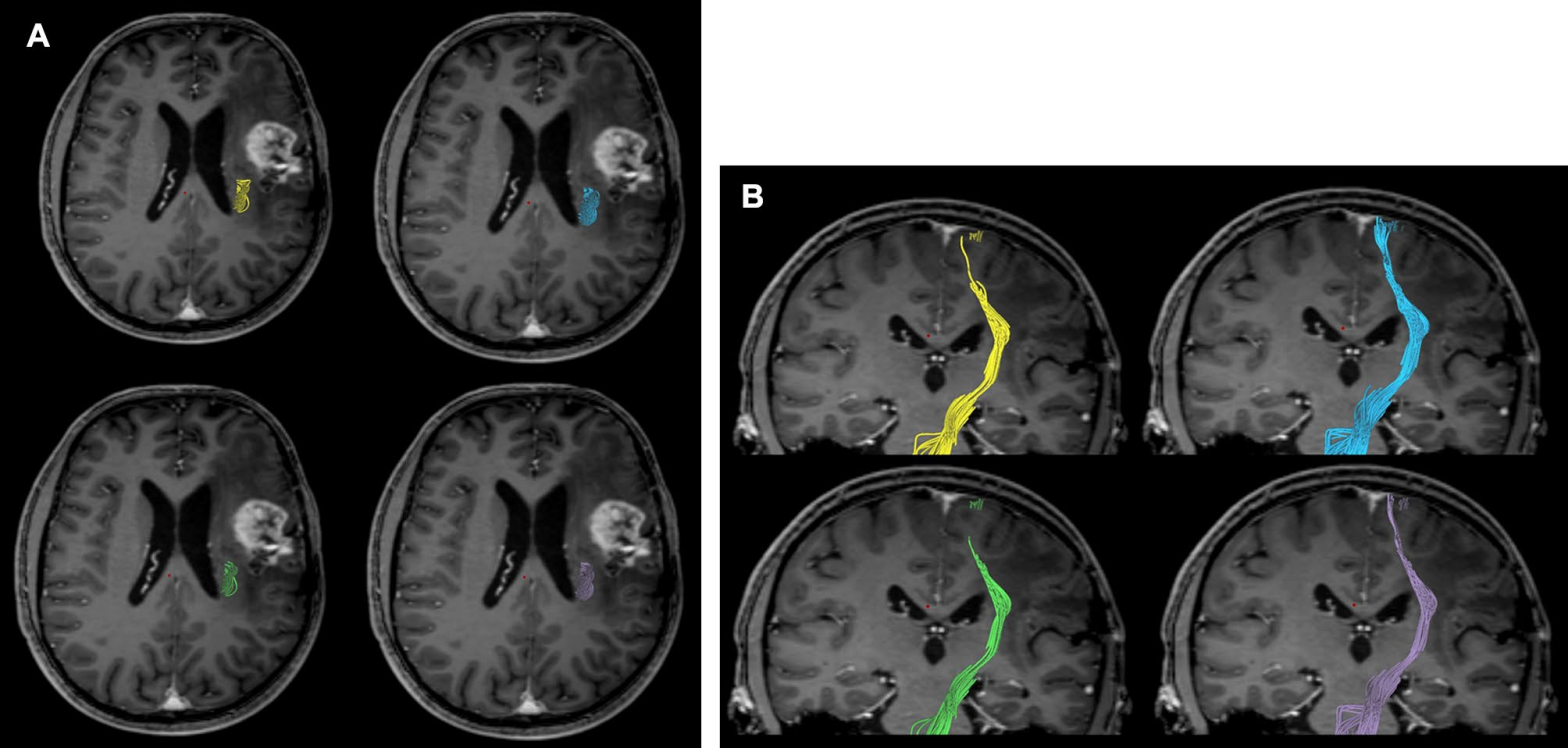
(**A**) Anatomic ROI based tractography at 50% FAT (yellow) and 75% FAT (green) and nTMS based tractography at 50% FAT (blue) and 75% FAT (purple). Axial views are displayed in (**A**), coronar views in (**B**) of the same patient.

A post-hoc reconstruction of the leg fibers of the corticospinal tract (CST) was performed using the Medtronic StealthStation S8® StealthViz DTI Module (Medtronic Inc. Louisville, CO, USA). For that purpose, the positive nTMS spots from the TA were exported from the NexStim software via the standard DICOM format in three different stimulation depths between 20 and 30 mm and imported into the tractography software. The nTMS spots were enlarged to a diameter of 6 mm and defined as a 3D object (Fig. 2A,B). This object was used as a cortical region of interest (ROI). A second ROI was defined within the inferior pons using the software’s manual drawing tool. Additionally, the leg fibers of the CST were visualized using a conventional anatomical approach blinded to the nTMS based ROI with a manual ROI within the suspected cortical site of the leg motor area near the interhemispheric fissure (Fig. 3A,B).

The seeding density was set to maximum, the maximum angular change to 45° and the fractional anisotropy (FA) was set to 75 and 50% of the maximum FA that allowed the visualization of fibers (FA-threshold—FAT). Minimum fiber length was set to 110 mm. Fibers which obviously did not belong to the corticospinal tract were removed. FAT, numbers of fibers and distances to the tumor were compared between the different settings.

### Decision making process and surgical workflow

Based upon the results of the whole nTMS examination of upper and lower extremity muscles, a multidisciplinary decision was made in each patient case. In six cases, the surgical strategy was changed from resection towards stereotactic biopsy or watch-and-wait. All surgeries were performed under general anesthesia. Intraoperative electrophysiological mapping using direct cortical bipolar stimulation (Nicolet EDX, Natus, WI, USA) was used upon the surgeon’s decision. The 3D MRI with additional information of the nTMS derived motor maps and tractography was used for neuronavigation, functional imaging data were implemented into the microscope on demand.

### Statistical Analysis

Statistical analysis was performed using IBM SPSS Version 27.0 (IBM SPSS Armonk, NY: IBM Corp) for Microsoft Windows. Continuous variables are presented as mean ± standard deviation (SD) and median ± interquartile range (IQR) if not otherwise declared. Categorial variables are presented as absolute number (n) and percent (%). We used the Mann–Whitney-U-Test and the Kruskal–Wallis-Test for continuous variables and the Fisher Exact Test and the Fisher-Freeman-Halton Test for categorical variables. Cramer’s V, odds ratios (OR) and the 95%-confidence interval (95% CI) are provided.

A *p* value of < 0.05 in two-tailed testing was considered statistically significant.

## Results

### Patient cohort

48 nTMS examinations were conducted in 46 different patients between April 2016 and September 2020. Two patients were mapped prior to primary and recurrent tumor removal. Mean age at examination was 57.27 ± 12.23 years, median 60.0 ± 15.75, and 19 (39.6%) of the examined patients were female. 11 patients (22.9%) presented with recurrent tumors. There was no histologically confirmed diagnosis in two cases (watch-and-wait strategy), however imaging was highly suspicious for low-grade gliomas.

Preoperatively, 22 patients (45.8%) presented with a motor-impairment. 7 (14.6%) presented with hemiparesis, isolated arm or leg paresis was present in 3 (6.3%) cases each, disturbance of fine motor skills was present in 8 patients (16.7%) and one patient (2.1%) had an isolated facial paresis. No patient suffered from upper or lower limb plegia and all but one patients were able to walk at the time of examination. Antiepileptic drug intake was documented in 25 patients (52.1%). Levetiracetam (1000-3000 mg/day) was used as a monotherapy in all but three patients. In the remaining patients where Carbamazepine (600 mg/day) or a combination of Levetiracetam and Valproate and an unknown combination therapy was applied. Antiepileptic drug intake was further divided into monotherapy low dose (Levetiracetam up to 1500 mg/day, Carbamazepine 600 mg/day) and higher dose (Levetiracetam 2000–3000 mg/day and combination therapy). Patient characteristics are summarized in Table 1.

**Table 1:** Overview of the whole patient cohort.

Item	N (%)	Mean (SD)
Age		57.27 ± 12.23
Female	19 (39.6%)	
**Motor status**		
Hemiparesis	7 (14.6%)	
Upper extremity paresis	3 (6.3%)	
Lower extremity paresis	3 (6.3%)	
Disturbance of fine motor skills	8 (16.7%)	
Antiepileptic drugs	25 (52.1%)	
Antiepileptic drugs lower dose	12 (25%)	
Antiepileptic drugs higher dose	12 (25%)	
Antiepileptic drugs unknown	2 (4.2%)	
**Tumor entity**		
Glioblastoma CNS WHO grade 4	21 (43.8%)	
Astrocytoma CNS WHO grade 3	4 (8.3%)	
Astrocytoma CNS WHO grade 2	4 (8.3%)	
Other primary brain tumors	4 (8.3%)	
Metastasis	15 (31.3%)	
Recurrent tumor	11 (22.9%)	
**Tumor location**		
Frontal	8 (16.7%)	
Parietal	10 (20.8%)	
Temporal	6 (12.5%)	
Gyrus precentralis	15 (31.3%)	
Gyrus postcentralis	9 (18.8%)	
Left-sided	23 (47.9%)	
Tumor volume (cm^3^)		20.15 ± 12.15
Midline shift	12 (25.0%)	
Edema within gyrus precentralis	19 (39.6%)	

### Results of navigated transcranial magnetic stimulation examinations

At least one MEP could be elicited in 27 patients (56.25%) and mapping of the TA muscle was considered reproducible in 20 (41.67%) of all patients. The mean number of applied stimulations within the anatomic leg motor area was 47.94 ± 34.54, median 39.5 ± 37.5. The mean number of MEPs was 13.3 ± 13.9, median 8.5 ± 10.0, per examination in patients with reproducible MEPs from the TA muscle, see Tables 2 and 3 for details. The lowest stimulation intensity to elicit MEPs was 146.8 ± 33.35, median 140.81 ± 60.25%rMT and ranged from 110.71 to 208.33%rMT. The highest stimulation intensity which elicited MEPs was 161.62 ± 42.69, median 160.62 ± 67.0%rMT. Stimulation intensity was increased in 40 examinations (83.3%) and ranged from a mean minimum intensity of 129.89 ± 20.06, median 126.6 ± 27.34%rMT, to a mean maximum intensity of 162.44 ± 42.67, median 151,47 ± 53.19%rMT.

**Table 2:** Comparison of patients with reproducible leg-MEPs and patients without reproducible leg-MEPs. For continuous variables, means and standard deviations are displayed.

	TA-MEPs reproducible (n = 20)	TA-MEPs not reproducible (n = 28)	p-value	Cramer's V	Odds Ratio	95% CI
Age	**52.8 ± 12.9**	**60.5 ± 10.8**	**0.044**			
Female	9 (45%)	10 (35.7%)	0.56	0.09	0.68	0.21–2.19
Lower limb paresis	3 (15%)	7 (25%)	0.49	0.12	0.53	0.12–2.36
Tumor entity			0.36	0.22		
Tumor recurrence	5 (25%)	6 (21.4%)	1.0	0.04	1.22	0.32–4.74
Tumor location			0.52	0.26		
Tumor volume (cm^3^)	25.9 ± 22.1	16.04 ± 17.18	0.17			
Midline shift	5 (25%)	7 (25%)	1.0	< 0.01	1.0	0.27–3.76
Edema within gyrus precentralis	**4 (20%)**	**15 (53.6%)**	**0.035**	**0.34**	**0.22**	**0.06–0.81**
Resting Motor Threshold (%)	33.5 ± 8.7	33.39 ± 13.07	0.46			
Minimum intensity to elicit MEPs/Maximum stimulation intensity (%rMT)	146.8 ± 33.35	162.56 ± 43.95	0.158			
Number of stimulations to leg area	52.3 ± 29.98	44.82 ± 37.68	0.2			
Number of MEPs	13.3 ± 13.9					

Significant values are in bold.

**Table 3:** Overview of nTMS stimulation parameters of the whole cohort, latencies, amplitudes and hotspot location are displayed for patients with reproducible MEPs from the TA muscle.

Item	Mean ± SD	Median ± IQR
Resting Motor Threshold (% stimulator output)	33.42 ± 11.33	31.0 ± 11.25
Stimulation intensity with a positive leg answer or maximum intensity (%rMT)	155.99 ± 40.26	146.09 ± 55.36
Number of stimulations to leg area	47.94 ± 34.54	39.5 ± 37.5
Latency (ms)	32.7 ± 3.22	32.81 ± 4,63
Amplitude (µV)	165.52 ± 87.77	139.2 ± 102.25

Hotspot location	N	%
Dorsal part of Superior Frontal Gyrus	5	25
Ventral part of Precentral Gyrus	9	45
Middle part of Precentral Gyrus	4	20
Dorsal margin of Precentral Gyrus	2	10

Patients with a motor deficit of their lower limbs received more stimulations (*p* = 0.021), but the distribution of the relative intensities did not differ. The results of nTMS examinations confirmed suspected infiltration to the leg motor area (*p* = 0.004, Cramer’s V = 0.84, OR = 0.06, 95%CI = 0.009–0.39). nTMS positive spots at tumor margin were further associated with leg motor deficits in patients with reproducible MEPs (*p* = 0.046, Cramer’s V = 0.61, OR = 32.0, 95%CI = 1.39–737.46).

### Influence of patient- and tumor-specific factors

Gender (*p* = 0.56, Cramer’s V = 0.09, OR = 0.68, 95% CI = 0.21–2.19) was not associated with reproducible MEPs from the TA muscle. Patients with reproducible MEPs from the TA were younger (*p* = 0.044).

Presence of a leg motor impairment was not associated with reproducible MEPs (*p* = 0.49, Cramer’s V = 0.12, OR = 0.53, 95%CI = 0.12–2.36).Tumor location (*p* = 0.52, Cramer’s V = 0.26), tumor entity (*p* = 0.36, Cramer’s V = 0.22), tumor recurrence (*p* = 1.0, Cramer’s V = 0.04, OR = 1.22, 95%CI = 0.32–4.74), left-sided tumor (*p* = 0.15, Cramer’s V = 0.22, OR = 0.4, 95%CI = 0.12–1.32) and tumor volume (*p* = 0.17) could be eliminated as factors which influenced the possibility to reproducibly elicit MEPs from the TA muscle. Edema within the motor system significantly reduced the possibility to elicit reproducible MEPs from the TA muscle (*p* = 0.035, Cramer’s V = 0.34, OR = 0.22, 95%CI = 0.06–0.81). Antiepileptic drug intake did not influence the feasibility to successfully map the TA muscle representation (*p* = 0.77, Cramer’s V = 0.06, OR = 0.8, 95%CI = 0.25–2.6) and the administered doses did further not influence the feasibility to reproducibly elicit MEPs (*p* = 0.28, Cramer’s V = 0.25).

### Influence of nTMS stimulation parameters

To rule out a learning effect, we compared the examination results of the different examination years with no differences concerning number of stimulations (*p* = 0.39), rMT (*p* = 0.185) and maximum stimulation intensity (*p* = 0.27) or the distribution of stimulation intensities (all *p* > 0.09). Additionally, the number of positive stimulations did not differ (*p* = 0.57). However, we found differences in stimulation intensity (*p* = 0.008) and total number of MEPs (*p* = 0.046) but not in total number of applied stimulations (*p* = 0.17) when different nTMS users (physician or medical student) were compared. Yet, there was no difference in the possibility to elicit MEPs reproducibly (*p* = 0.053, Cramer’s V = 0.3, OR = 0.18, 95%CI = 0.03–1.0).

**Figure 4:**
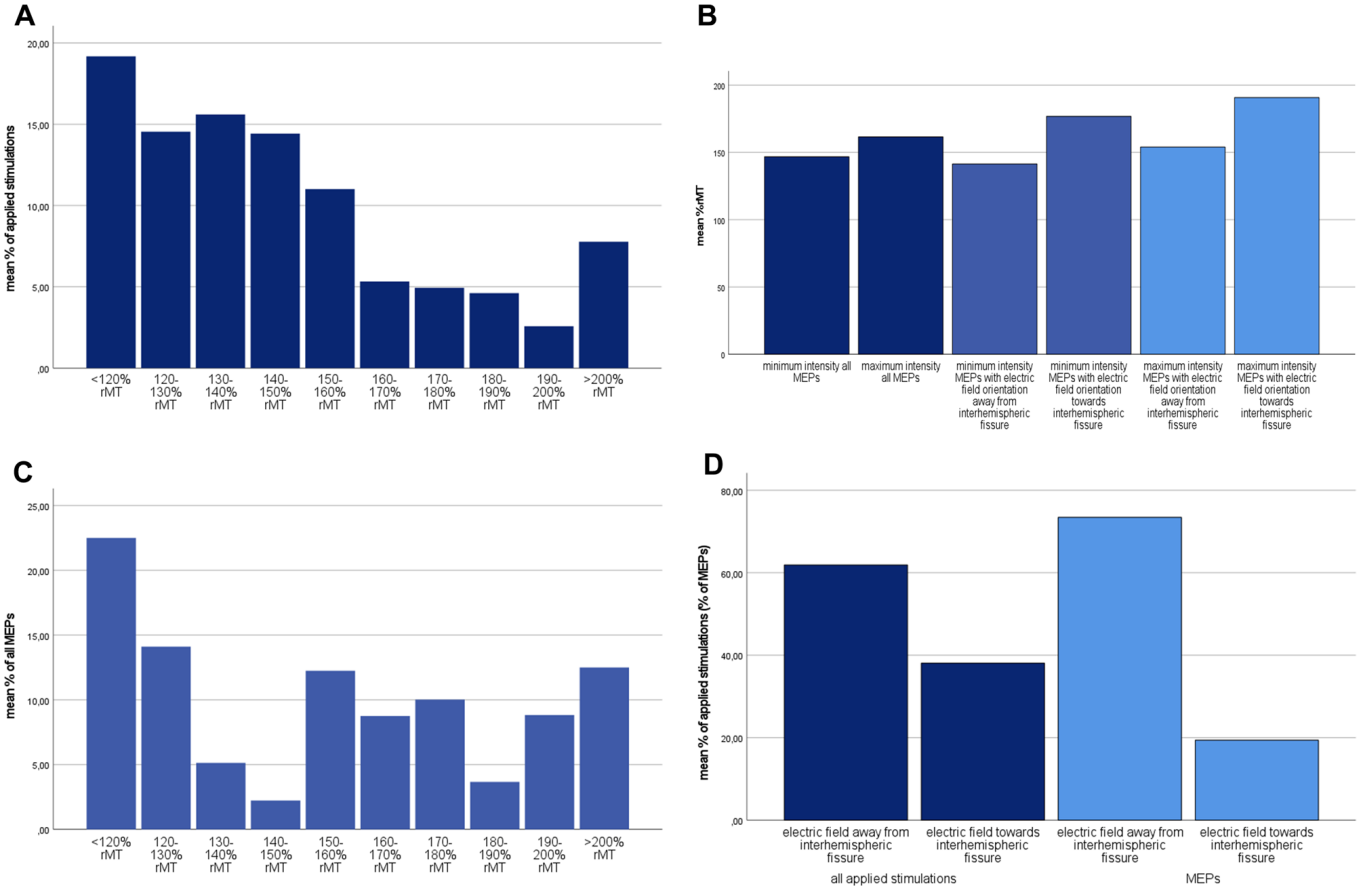
(**A**) Relative amount of applied stimulations at different intensities in the whole patient cohort. Please note that not every patient received stimulations at each intensity step. (**B)** Mean intensities at which MEPs were registered: mean lowest intensity, mean maximum intensity, the mean minimum and maximum intensities with different orientations of the electric field. The minimum intensity to elicit MEPs was lower when the electric field was orientated away from the interhemispheric fissure (*p* = 0.022). (**C**) Relative amount of MEPs at different stimulation intensities in patients with reproducible MEPs. (**D**) distribution of all stimulations and MEPs at each electric field direction in patients with reproducible MEPs. Total amount of stimulations was not different, whereas the MEP counts showed a significant difference in favor of the main electric field direction pointing away from the interhemispheric fissure (*p* = 0.01).

In patients with reproducible MEPs, maximum applied stimulation intensity (*p* = 0.158), resting motor threshold (*p* = 0.46) and total number of applied stimulations (*p* = 0.2) were not statistically different compared to patients with no reproducible MEPs from the TA. The increase in stimulation intensity from the lowest applied intensity to either the lowest intensity to elicit MEPs or the maximum applied intensity did also not differ (*p* = 0.14). Figure 4A–D illustrates the applied stimulation intensities at which MEPs were registered and the distribution of applied stimulations at different stimulation intensities as well as the relative amounts of MEPs elicited at different stimulation intensities.

The number of stimulations with electric field orientation towards the interhemispheric fissure or towards the hemisphere of interest was not different if MEPs could reproducibly be elicited or not (towards the interhemispheric fissure* p* = 0.59, towards the hemisphere of interest *p* = 0.29). Additionally, there were no significant differences in number of applied stimulations at each electric field direction when different stimulation intensities were evaluated if mapping was conducted successfully or not (all *p* > 0.17). However, in patients with reproducible MEPs, the direction of the electric field towards the hemisphere of interest elicited a higher number of MEPs (*p* = 0.01) at lower intensities (*p* = 0.022) with no differences in total number of applied stimulations (*p* = 0.45). (Fig. 4D).

Edema involving the precentral gyrus did not lead to different stimulation parameters (maximum stimulation intensity *p* = 0.39, number of stimulations *p* = 0.41).

Yet, perifocal edema within the precentral gyrus was associated with tumor infiltration into the precentral gyrus (*p* = 0.013, Cramer’s V = 0.37, OR = 5.33, 95%CI = 1.43–19.94).

Mean MEP-latencies (*p* = 0.62) and amplitudes (*p* = 0.69) did not differ if the tumor infiltrated the nTMS positive leg area or if patients suffered from a leg motor impairment (*p* = 0.92 for mean latency, *p* = 0.92 for mean amplitude, respectively). Edema did not influence amplitudes (*p* = 0.55) and latencies (*p* = 0.55). In contrast, the intake of higher doses of antiepileptic drugs lowered the mean amplitudes (*p* = 0.017), but not the mean latency (*p* = 0.85). Maximum applied stimulation intensities did not differ in patients with higher doses of antiepileptic drugs (*p* = 0.23).

### Tractography analysis

DTI-sequences were available in 39 cases (81.25%) and in 15 patients with reproducible MEPs of the TA (75%). In one patient the predefined tractography settings did not visualize any fibers in the anatomical approach. This was also the case for two patients for nTMS based tractography.

The mean fractional anisotropy threshold (FAT) for nTMS based fibertracking was 0.33 ± 0.12, median 0.37 ± 0.19. Mean number of fibers was 23.85 ± 64.02, median 4.0 ± 12.0, at 75% FAT and 43.96 ± 98.94, median 7.0 ± 29.0, at 50% FAT. The mean distance between the tumor and the margin of the fibers was 16.82 ± 12.89 mm, median 11.6 ± 22.0 mm, at 75% FAT and 14.96 ± 13.24 mm, median 8.5 ± 25.55 mm, at 50% FAT. The tractography results are outlined in Tables 4 and 5 and illustrated in Fig. 3.

**Table 4:** Tractography of patients with reproducible TA-MEPs.

Tractography analysis (patients with reproducible TA-MEPs, n = 15)	nTMS based fibertracking (n = 13)		Anatomic ROI based fibertracking (n = 15)		
Item	Mean (SD)	Median (IQR)	Mean (SD)	Median (IQR)	*p* value
FA-threshold	0.33 ± 0.12	0.37 ± 0.19	0.44 ± 0.12	0.47 ± 0.13	0.025
**Fibertracking at 50%FAT**					
Distance lesion to CST (mm)	14.96 ± 13.24	8.5 ± 25.55	12.93 ± 13.5	6.0 ± 22.0	0.68
Number of fibers (n)	43.96 ± 98.94	7.0 ± 29.0	56.3 ± 67.6	34.0 ± 26.0	0.072
**Fibertracking at 75%FAT**					
Distance lesion to CST (mm)	16.82 ± 12.89	11.6 ± 22.0	14.73 ± 12.61	9.2 ± 18.5	0.72
Number of fibers (n)	23.85 ± 64.02	4.0 ± 12.0	27.47 ± 35.05	14.0 ± 28.0	0.094

**Table 5:** Comparison of anatomic based tractography of patients with reproducible MEPs and patients in which mapping of the TA muscle was not possible or reproducible.

Tractography analysis (anatomic-ROI-based fibertracking)	TA-MEPs reproducible		TA-MEPs not possible/repoducible		
Item	Mean (SD)	Median (IQR)	Mean (SD)	Median (IQR)	*p *value
FA-threshold	0.44 ± 0.12	0.47 ± 0.13	0.44 ± 0.19	0.44 ± 0.34	0.91
**Fibertracking at 50%FAT**					
Distance lesion to CST (mm)	12.93 ± 13.5	6.0 ± 22.0	5.2 ± 8.6	1.8 ± 6.9	0.014
Number of fibers (n)	56.3 ± 67.6	34.0 ± 26.0	196.73 ± 359.53	79.0 ± 118	0.033
**Fibertracking at 75%FAT**					
Distance lesion to CST (mm)	14.73 ± 12.61	9.2 ± 18.5	6.73 ± 9.76	2.9 ± 9.1	0.005
Number of fibers (n)	27.47 ± 35.05	14.0 ± 28.0	74.83 ± 123.51	25.0 ± 32.0	0.033

The distance between the tumor and the pyramidal tract did not differ between the anatomy-based fiber tracking and the nTMS based fiber tracking at both FA-values (*p* = 0.72 for 75% FAT and *p* = 0.68 for 50% FAT). Tractography based upon an anatomic ROI allowed tracking at higher FA-values (*p* = 0.025), but did not visualize more fibers (*p* = 0.094 for 75% FAT; *p* = 0.072 for 50% FAT).

Concerning the whole cohort, the distance between the tumor and the pyramidal tract was higher in patients with reproducible MEPs from the TA muscle (*p *= 0.005 for 75% FAT, *p* = 0.014 for 50% FAT) at the anatomy based approach, see Table 5.

### Correlation with clinical findings

The distance between the lesion and the pyramidal tract was shorter for all anatomy-based CST-reconstructions if the patient suffered preoperatively from a tumor-induced leg motor impairment (*p* = 0.004 at 75%FAT, *p* = 0.001 at 50%FAT, respectively). Intraoperative mapping with direct cortical stimulation of arm and leg muscles was used in 17 procedures (40.5%). Intraoperative stimulation was used in patients with closer distances between the CST and the lesion (*p* = 0.012 for the anatomy-based approach, *p* > 0.07 for the nTMS-based approach). The usage of intraoperative mapping was not associated with the possibility to map the leg motor area with nTMS successfully (*p* = 0.21, Cramer’s V = 0.22, OR = 0.39, 95%CI = 0.1–1.42).

At discharge from hospital (mean 9.38 ± 5.34, median 8 ± 3 days postoperatively), leg motor function deteriorated in 9 cases (18.8%) and improved in 2 patients (4.2%) compared to the preoperative status. We did not observe an association between the possibility to elicit reproducible MEPs from the TA and postoperative deterioration in leg function (*p* = 0.26, Cramer’s V = 0.22, OR = 0.3, 95%CI = 0.06–1.68). Additionally, there was no association between the usage of intraoperative mapping and postoperative deterioration in motor function in general (p = 0.19, Cramer’s V = 0.21, OR = 2.5, 95%CI = 0.66–9.46) or deterioration in leg function (*p* = 0.7, Cramer’s V = 0.1, OR = 1.6, 95%CI = 0.36–7.18).

However, regarding motor outcome in general, we discovered a statistical association between the aggravation of preoperative deficits and the lack of eliciting reproducible TA-MEPs: no patient with a motor impairment and reproducible TA-MEPs further deteriorated postoperatively (*p* = 0.005, Cramer’s V = 0.45, OR = 0.63, 95%CI = 0.46–0.85).

We did observe greater distances between the tumor and the corticospinal tract if motor function in general remained stable postoperatively (*p* = 0.002 for 75%FAT anatomic ROI-seeding, *p* = 0.033 for 50% FAT anatomic ROI-seeding, respectively). However, we did not observe shorter distances to the CST in patients with postoperative deterioration in leg motor function (all *p* > 0.072).

## Discussion

Preoperative motor mapping with navigated transcranial magnetic stimulation has changed decision making in brain tumor patients with motor-eloquent lesions and several studies could demonstrate a prognostic benefit of patients who received preoperative brain mapping as well as distinct advantages in the presurgical planning process^3,4,14,16,23–37^. In the clinical setting, preoperative information regarding the cortical leg motor representation seems utmost important in patients with malignant tumors close to the falx cerebri as well as even meningiomas located at the falx cerebri^37^.

In our study, we outlined influencing factors of nTMS mapping of the leg motor area in the field of neurosurgical mapping and planning. The aim of our study was to determine factors which influence the possibility and quality of evoking MEPs from the TA muscle to produce reproducible maps of the cortical leg area suitable for neurosurgical planning in the clinical course of brain tumor patients. The main factors which influence the results of nTMS leg mapping were patient age, perifocal edema within the motor cortex and distance of the lesion to the corticospinal tract.

In our study, stimulation intensity was no significant factor which facilitated leg mapping. However, there was a disparity concerning the applied stimulation intensities among different examiners. Additionally, the examiner was a non-significant factor for the possibility to elicit MEPs at all. In previously published studies, leg mapping was possible in 50–90% of the participants, which is in unison with our results^15–17,38^. Studies could demonstrate both difficulties in mapping the leg area in healthy subjects and in patients with lower limb post-stroke palsy concerning both feasibility and reproducibility^16,38^.

The results of the present study suggest that leg motor mapping is influenced by several factors and is not purely dependent on stimulation parameters such as stimulation intensity, resting motor threshold or number of stimulations. Younger age was associated with a higher chance for successful leg mapping. Motor function seemed to be an obvious confounder since applying stimulations to a paretic extremity is a known influencing factor to the rMT and MEP latency^5,6,38^. In our study, motor deficits did not influence the feasibility of leg mapping with nTMS. Nevertheless, motor deficit is a known factor to influence nTMS mapping parameters^6^, but nTMS mapping is possible in patients with motoric deficits of the mapped extremities^38,39^. However, the distance between the tumor and the leg fibers significantly influenced the feasibility to elicit MEPs indicating that a slight disturbance of the motor system decreases the chance to create a motor map for the TA muscle and the CST-reconstructions correlated well with the preoperative motor status. As stated above, peritumoral edema involving the motor system was one main factor which prohibited MEPs from the TA muscle. The impact of vasogenic or peritumoral brain edema on the excitability of neurons remains unclear but there are certain indications that edema might increase the excitability of neurons which is in contrast to our results^5,6^.

Based upon the results of the presented study and previously published other cohorts^17,20,40,41^, the question arises if it would be sensible to apply slight adjustments to the mapping algorithm. In our study, there was a wide range of applied stimulation intensities and subsequently a wide range of stimulation intensities at which MEPs could be elicited. The lowest intensity which elicited MEPs was 110.71%rMT which is the intensity at which mapping of the hand motor area is usually performed^2,4–6,14,20,21,33–35,40,42–44^. However, the mean intensity to elicit MEPs from the TA was higher in most patients.

Increasing the stimulator output is recommended to elicit the first leg-MEP^20^. In non-parametric-testing the stimulation intensities did not differ in patients with or without positive leg responses.

In many published nTMS studies, the lower extremity muscles were mapped with at least 130% of the patient-individual resting motor threshold of the upper extremity^15,34,45^. The motor thresholds of the lower extremity muscles seem to be higher and the excitability of the lower limb muscles was found to be of prognostic value^40,41,46^. It seems sensible to separately determine the rMT for the lower limb in the clinical routine setting. We would recommend to start mapping at 110%rMT and increase the stimulation intensity in steps of 10–20%rMT in case of no MEPs after 15–20 stimulations in cases of no MEPs. At an intensity at which mapping the leg area is reproducibly possible, the hotspot of the leg motor area could be determined and a separate rMT for the leg muscles could be established in order to perform a second round of mapping at a distinct supra-threshold intensity for the leg muscles, e.g. 105–110%rMT of the TA. The main electric field direction should point towards the hemisphere of interest^20^. In cases of no MEPs, the chance of recording a single MEP should be raised with a higher number of monitored muscles due to an increased cortical target area and possible overlap in cortical representations of lower limb muscles together with decreased focality in stimulation of the lower limb muscles and higher stimulation intensities^10,47–49^ Having the patient pre-activated the targeted muscle is further recommended in cases of no MEPs^20^, but pre-activation was not performed in our investigation. We might therefor underestimate the rate of patients with successful mapping in our cohort.

Changing the stimulation protocol from single to paired pulse-nTMS might further improve the quality of leg mapping resulting in more robust maps in combination with lower stimulation intensities when using paired-pulse-nTMS^17,50,51^. In our study, only one single MEP was registered in 14.6% of the examinations. We considered these MEPs as not sufficient for surgical planning, since one MEP is not suitable as a motor map. In these cases, we would recommend to extend the nTMS examination as stated above in order to be able to record several MEPs and to gather more information on the cortical leg muscle representations. We would further recommend to use intraoperative stimulation to verify the results of the nTMS examination in unclear situations since the accuracy of nTMS correlated well with intraoperative mapping^14,28,40^.

We conclude that the possibility of eliciting MEPs from the TA muscle might follow an “all-or-nothing-rule” and the TA muscle of some patients is easier to stimulate. One answer might be the cortical representation of the lower limb at the interhemispheric fissure. The alignment of the cortical neurons there is different compared to the hand motor hotspot. This leads to an altered orientation of the electric field of the nTMS coil. This change in geometry might lower the chances to stimulate these neurons with the rather focal figure-of-eight-coil.

The results of our study also indicate a prognostic value of the possibility to create a motor map of the lower limb as patients were more likely to deteriorate if it was not possible to record MEPs from the TA muscle. These results need to be interpreted with respect to the distance between the tumor and the pyramidal tract which was shorter in patients who deteriorated postoperatively.

## Limitations and implications for further research

Although the study design is retrospective, the presented study has several strengths. The examination protocol was highly standardized ensuring comparability of the examinations. The stimulation intensities did not vary significantly and there was no significant difference in the total number of applied stimulations among the whole cohort. However, the stimulation intensity was not elevated to the maximum stimulator output of the nTMS system in cases with no MEPs to be recorded. This might underestimate the rates of possible MEPs. Further, we did not evaluate different lower extremity muscles or several simultaneously monitored muscles, but results of TMS-examinations in healthy subjects suggest a high overlap in cortical leg representation^10^.

Another limitation is that we only examined the hemisphere that was affected by the tumor, which might lead to an underestimation of patient-specific factors like age or gender. However, in presurgical nTMS motor mapping, information concerning the affected hemisphere are most important when aiming at the best achievable result for the particular patient. Future research should be conducted to compare the excitability of both hemispheres especially in mapping the leg area in order to increase the amount of knowledge concerning the influence of patient-individual factors in the context of mapping the leg area with nTMS. Further, our results only apply to brain tumor patients since one main reason for successful mapping in our cohort was the absence of perilesional vasogenic edema which is usually not present in non-tumor patients who receive nTMS examinations for other diagnostic or therapeutic reasons.

## Conclusion

Mapping the leg area with navigated transcranial magnetic stimulation is challenging for the nTMS user in neuro-oncologic patients. One main reason for these difficulties might be the anatomy and geometry of the cortical areas within the interhemispheric fissure. The main factors for successful mapping of the tibialis anterior muscle were absence of peritumoral edema and younger patient age. nTMS leg-mapping cannot be solely facilitated with an increase of stimulation intensity. Electric field orientation was no significant factor within the constraint of the predefined coil orientation.

## Data Availability

The datasets which support the findings of this study are available from the corresponding author upon reasonable request.

## Abbreviations

DICOM: Digital Imaging and Communications in Medicine
DTI: Diffusion tensor imaging
EMG: Electromyogram
FA: Fractional anisotropy
FAT: Fractional anisotropy-threshold
MEP: Motor evoked potential
MRI: Magnetic resonance imaging
nTMS: Navigated transcranial magnetic stimulation
rMT: Resting motor threshold
ROI: Region of interest
TA: Tibialis anterior (muscle)

## References

[1] Krings T (2001). Introducing navigated transcranial magnetic stimulation as a refined brain mapping methodology. Neurosurg. Rev..

[2] Frey D (2014). Navigated transcranial magnetic stimulation improves the treatment outcome in patients with brain tumors in motor eloquent locations. Neuro Oncol..

[3] Picht T (2012). Assessment of the influence of navigated transcranial magnetic stimulation on surgical planning for tumors in or near the motor cortex. Neurosurgery.

[4] Krieg SM (2015). Changing the clinical course of glioma patients by preoperative motor mapping with navigated transcranial magnetic brain stimulation. BMC Cancer.

[5] Sollmann N (2017). The variability of motor evoked potential latencies in neurosurgical motor mapping by preoperative navigated transcranial magnetic stimulation. BMC Neurosci..

[6] Sollmann N (2017). Clinical factors underlying the inter-individual variability of the resting motor threshold in navigated transcranial magnetic stimulation motor mapping. Brain Topogr..

[7] Niskanen E (2010). Group-level variations in motor representation areas of thenar and anterior tibial muscles: navigated transcranial magnetic stimulation study. Hum. Brain Mapp..

[8] Sollmann N (2013). Inter- and intraobserver variability in motor mapping of the hotspot for the abductor policis brevis muscle. BMC Neurosci..

[9] Al Sawah M (2014). Symmetric corticospinal excitability and representation of vastus lateralis muscle in right-handed healthy subjects. Clin. Anat..

[10] Davies JL (2020). Using transcranial magnetic stimulation to map the cortical representation of lower-limb muscles. Clin. Neurophysiol. Pract..

[11] Ward S (2016). Cortical motor representation of the rectus femoris does not differ between the left and right hemisphere. J. Electromyogr. Kinesiol..

[12] Sivaramakrishnan A, Tahara-Eckl L, Madhavan S (2016). Spatial localization and distribution of the TMS-related 'hotspot' of the tibialis anterior muscle representation in the healthy and post-stroke motor cortex. Neurosci. Lett..

[13] Picht T (2009). Navigated transcranial magnetic stimulation for preoperative functional diagnostics in brain tumor surgery. Neurosurgery.

[14] Picht T (2011). Preoperative functional mapping for rolandic brain tumor surgery: comparison of navigated transcranial magnetic stimulation to direct cortical stimulation. Neurosurgery.

[15] Krieg SM (2012). Utility of presurgical navigated transcranial magnetic brain stimulation for the resection of tumors in eloquent motor areas. J. Neurosurg..

[16] Weiss C (2013). Mapping the hand, foot and face representations in the primary motor cortex - retest reliability of neuronavigated TMS versus functional MRI. Neuroimage.

[17] Zhang H (2020). Short-interval intracortical facilitation improves efficacy in nTMS motor mapping of lower extremity muscle representations in patients with supra-tentorial brain tumors. Cancers (Basel).

[18] Ahdab R, Ayache SS, Brugieres P, Farhat WH, Lefaucheur JP (2016). The hand motor hotspot is not always located in the hand knob: a neuronavigated transcranial magnetic stimulation study. Brain Topogr..

[19] Hand BJ, Opie GM, Sidhu SK, Semmler JG (2020). TMS coil orientation and muscle activation influence lower limb intracortical excitability. Brain Res..

[20] Krieg SM (2017). Protocol for motor and language mapping by navigated TMS in patients and healthy volunteers; workshop report. Acta Neurochir. (Wien).

[21] Sollmann N (2018). Associations between clinical outcome and navigated transcranial magnetic stimulation characteristics in patients with motor-eloquent brain lesions: a combined navigated transcranial magnetic stimulation-diffusion tensor imaging fiber tracking approach. J. Neurosurg..

[22] Säisänen L (2008). Motor potentials evoked by navigated transcranial magnetic stimulation in healthy subjects. J. Clin. Neurophysiol..

[23] Rosenstock T (2017). Specific DTI seeding and diffusivity-analysis improve the quality and prognostic value of TMS-based deterministic DTI of the pyramidal tract. Neuroimage Clin..

[24] Rosenstock T (2017). Risk stratification in motor area-related glioma surgery based on navigated transcranial magnetic stimulation data. J. Neurosurg..

[25] Rosenstock T (2021). Bicentric validation of the navigated transcranial magnetic stimulation motor risk stratification model. J. Neurosurg..

[26] Rosenstock T (2021). Preoperative nTMS and intraoperative neurophysiology—a comparative analysis in patients with motor-eloquent glioma. Front. Oncol..

[27] Weiss Lucas C (2022). Surgery of motor eloquent glioblastoma guided by TMS-informed tractography: driving resection completeness towards prolonged survival. Front. Oncol..

[28] Weiss Lucas C (2020). Invasive versus non-invasive mapping of the motor cortex. Hum. Brain Mapp..

[29] WeissLucas C (2017). Functional MRI vs. navigated TMS to optimize M1 seed volume delineation for DTI tractography. A prospective study in patients with brain tumours adjacent to the corticospinal tract. Neuroimage Clin..

[30] Hendrix P (2020). Preoperative navigated transcranial magnetic stimulation improves gross total resection rates in patients with motor-eloquent high-grade gliomas: a matched cohort study. Neurosurgery.

[31] Hendrix P (2017). Preoperative navigated transcranial magnetic stimulation and tractography in transparietal approach to the trigone of the lateral ventricle. J. Clin. Neurosci..

[32] Hendrix P (2016). Preoperative navigated transcranial magnetic stimulation in patients with motor eloquent lesions with emphasis on metastasis. Clin. Anat..

[33] Krieg SM (2016). Resection of motor eloquent metastases aided by preoperative nTMS-based motor maps-comparison of two observational cohorts. Front. Oncol..

[34] Krieg SM (2014). Preoperative motor mapping by navigated transcranial magnetic brain stimulation improves outcome for motor eloquent lesions. Neuro Oncol..

[35] Picht T, Frey D, Thieme S, Kliesch S, Vajkoczy P (2016). Presurgical navigated TMS motor cortex mapping improves outcome in glioblastoma surgery: a controlled observational study. J. Neurooncol..

[36] Picht T, Schulz J, Vajkoczy P (2013). The preoperative use of navigated transcranial magnetic stimulation facilitates early resection of suspected low-grade gliomas in the motor cortex. Acta Neurochir. (Wien).

[37] Raffa G (2019). Surgical treatment of meningiomas located in the rolandic area: the role of navigated transcranial magnetic stimulation for preoperative planning, surgical strategy, and prediction of arachnoidal cleavage and motor outcome. J. Neurosurg..

[38] Peters HT (2017). navigated transcranial magnetic stimulation: A biologically based assay of lower extremity impairment and gait velocity. Neural Plast..

[39] Picht T, Schmidt S, Woitzik J, Suess O (2011). Navigated brain stimulation for preoperative cortical mapping in paretic patients: case report of a hemiplegic patient. Neurosurgery.

[40] Forster MT (2011). Navigated transcranial magnetic stimulation and functional magnetic resonance imaging: advanced adjuncts in preoperative planning for central region tumors. Neurosurgery.

[41] Lavrador JP (2020). Altered motor excitability in patients with diffuse gliomas involving motor eloquent areas: the impact of tumor grading. Neurosurgery.

[42] Forster MT, Limbart M, Seifert V, Senft C (2014). Test-retest reliability of navigated transcranial magnetic stimulation of the motor cortex. Neurosurgery.

[43] Picht T (2012). Assessing the functional status of the motor system in brain tumor patients using transcranial magnetic stimulation. Acta Neurochir. (Wien).

[44] Raffa G (2019). Multimodal surgical treatment of high-grade gliomas in the motor area: the impact of the combination of navigated transcranial magnetic stimulation and fluorescein-guided resection. World Neurosurg..

[45] Conti A (2014). Navigated transcranial magnetic stimulation for "somatotopic" tractography of the corticospinal tract. Neurosurgery.

[46] Rossini PM (2015). Non-invasive electrical and magnetic stimulation of the brain, spinal cord, roots and peripheral nerves: Basic principles and procedures for routine clinical and research application. An updated report from an I.F.C.N. Committee. Clin. Neurophysiol..

[47] Kallioniemi E, Julkunen P (2016). Alternative stimulation intensities for mapping cortical motor area with navigated TMS. Brain Topogr..

[48] Kesar TM, Stinear JW, Wolf SL (2018). The use of transcranial magnetic stimulation to evaluate cortical excitability of lower limb musculature: Challenges and opportunities. Restor. Neurol. Neurosci..

[49] van de Ruit M, Grey MJ (2016). The TMS map scales with increased stimulation intensity and muscle activation. Brain Topogr..

[50] Pitkanen M, Kallioniemi E, Jarnefelt G, Karhu J, Julkunen P (2018). Efficient mapping of the motor cortex with navigated biphasic paired-pulse transcranial magnetic stimulation. Brain Topogr..

[51] Sollmann N (2020). Paired-pulse navigated TMS is more effective than single-pulse navigated TMS for mapping upper extremity muscles in brain tumor patients. Clin. Neurophysiol..

